# Studies of Streptococcus anginosus Virulence in Dictyostelium discoideum and Galleria mellonella Models

**DOI:** 10.1128/iai.00016-23

**Published:** 2023-04-25

**Authors:** Joanna Budziaszek, Magdalena Pilarczyk-Zurek, Ewelina Dobosz, Aleksandra Kozinska, Dariusz Nowicki, Katarzyna Obszanska, Agnieszka Szalewska-Pałasz, Izabela Kern-Zdanowicz, Izabela Sitkiewicz, Joanna Koziel

**Affiliations:** a Department of Microbiology, Faculty of Biochemistry, Biophysics and Biotechnology, Jagiellonian University, Krakow, Poland; b Department of Drug Biotechnology and Bioinformatics, National Medicines Institute, Warsaw, Poland; c Department of Molecular Biology, University of Gdańsk, Gdańsk, Poland; d Institute of Biochemistry and Biophysics, Polish Academy of Sciences, Warsaw, Poland; e Institute of Biology, Warsaw University of Life Sciences-SGGW, Warsaw, Poland; University of Illinois Chicago

**Keywords:** *Streptococcus anginosus*, *Galleria mellonella*, *Dictyostelium discoideum*, virulence, infection models, neutrophils

## Abstract

For many years, Streptococcus anginosus has been considered a commensal colonizing the oral cavity, as well as the gastrointestinal and genitourinary tracts. However, recent epidemiological and clinical data designate this bacterium as an emerging opportunistic pathogen. Despite the reported pathogenicity of S. anginosus, the molecular mechanism underpinning its virulence is poorly described. Therefore, our goal was to develop and optimize efficient and simple infection models that can be applied to examine the virulence of S. anginosus and to study host-pathogen interactions. Using 23 S. anginosus isolates collected from different infections, including severe and superficial infections, as well as an attenuated strain devoid of CppA, we demonstrate for the first time that Dictyostelium discoideum is a suitable model for initial, fast, and large-scale screening of virulence. Furthermore, we found that another nonvertebrate animal model, Galleria mellonella, can be used to study the pathogenesis of S. anginosus infection, with an emphasis on the interactions between the pathogen and host innate immunity. Examining the profile of immune defense genes, including antimicrobial peptides, opsonins, regulators of nodulation, and inhibitors of proteases, by quantitative PCR (qPCR) we identified different immune response profiles depending on the S. anginosus strain. Using these models, we show that S. anginosus is resistant to the bactericidal activity of phagocytes, a phenomenon confirmed using human neutrophils. Notably, since we found that the data from these models corresponded to the clinical severity of infection, we propose their further application to studies of the virulence of S. anginosus.

## INTRODUCTION

Streptococcus anginosus, along with Streptococcus constellatus and Streptococcus intermedius, belongs to the Streptococcus anginosus group. Initially, this group was classified as the primary commensal of the mucosal membranes of the oral cavity, but it can also colonize the throat, nasopharynx, gastrointestinal tract, and genitourinary tract (1). Improved diagnostic methods revealed that this species of streptococcus has extensive pathogenic potential; indeed, a growing number of life-threatening infections have been documented in clinical practice. S. anginosus is frequently found in cases of bacteremia (2), liver abscess, and gastrointestinal and genitourinary tract infections (3). Although clinical data indicate that S. anginosus should be considered an opportunistic pathogen, few studies have examined its virulence strategies (3).

Data from available virulence and comparative genomic-sequencing studies revealed that S. anginosus encodes putative virulence factors common to other streptococci; these include hemolysins (4) adhesins (5), DNases (6), the capsule (7), toxins (8), pili (9), hyaluronidases (10), enolases (9), and superantigens (6). Although their role in the pathogenicity of S. anginosus has been postulated, experimental evidence is lacking. The majority of data that describe the role of putative determinants of S. anginosus virulence comes from *in vitro* studies (11, 12), while the real mechanisms underlying the bacterial interaction with the host remain unknown, primarily due to a lack of efficient animal models.

Dictyostelium discoideum is a simple, unicellular eukaryotic model system that has been developed to screen for bacterial virulence. The model has been exploited mainly to study the pathogenic potential of Gram-negative species, including Vibrio cholerae (13), Aeromonas hydrophila (14), Pseudomonas aeruginosa (15, 16), Burkholderia cenocepacia (17), and Burkholderia pseudomallei (18), as well as Gram-positive bacteria, such as Mycobacterium marinum (19). Among streptococcal species, only Streptococcus suis was examined in the described model (20). More importantly, a D. discoideum model provides the ability to track the infection process at the level of genetic and biochemical interactions using well-established tools (21–24). The vast availability of amoeba mutant strains allows the evaluation of pathogen-host interactions at the molecular level (25, 26).

Recently, lower vertebrates and invertebrates, such as Caenorhabditis elegans, Drosophila melanogaster, Danio rerio, and Galleria mellonella, became an attractive alternative to conventional murine models for studying host-pathogen interactions. They are used to examine the molecular mechanisms underlying the virulence strategies directed against the innate immune system and also to study the effectiveness of antimicrobial compounds *in vivo* (27). Among them are G. mellonella larvae, which exhibit immune defense mechanisms analogous to human innate responses, including both the cellular and the humoral components. There are at least eight types of hemocyte showing great similarity to the mammalian phagocytes that play a central role in antibacterial defense (28–30). The humoral response is achieved via the production of soluble effector molecules that immobilize or kill the pathogen. They include proteins homologous to the complement system, antibacterial peptides, and melanin (31, 32). The larvae can be used for subcutaneous injection, oral administration, and *in vivo* imaging (33, 34), for monitoring intracellular gene and protein expression (35–37), to detect immune responses (38), and to test antimicrobial drugs (39, 40). The use of this surrogate infectious model does not require special facilities; its short time and cost efficiency are significant benefits, and it does not raise ethical concerns. The lack of genetically modified strains of G. mellonella is a disadvantage, as the whole genome was described only in 2018 (41). Nevertheless, G. mellonella seems to be a very useful model for studying the corruption of innate immune mechanisms by pathogens.

The purpose of this study was to optimize two experimental models, D. discoideum and G. mellonella, in order to examine the virulence of S. anginosus. We found that D. discoideum can be used efficiently for large-scale screening of clinical isolates of S. anginosus with respect to their pathogenicity. To study the interactions between the bacteria and the host defense system and to explore the pathogenesis of S. anginosus infection, we used the G. mellonella model. We found during model development that S. anginosus strains varied in terms of their resistance to the bactericidal activity of phagocytes; therefore, the data obtained were verified using primary human neutrophils. Taken together, the data provide for the first time a set of models that can be used to investigate the mechanisms of S. anginosus pathogenesis.

## RESULTS

### Collection of Streptococcus anginosus isolates.

For this study, we used a clinical collection of 23 S. anginosus isolates obtained from cases of severe (bacteremia [*n* = 4] and abscess [*n* = 8]) and mild (skin wounds [*n* = 2] and pharyngitis [*n* = 9]) infection. These strains were phenotypically characterized by examining the type of the Lancefield antigen, the type of hemolysis (alpha, beta, or gamma), DNase activity, and the presence of the capsule [Table 1]. Given the different origins of the strains, as well as their varied phenotypes, we assumed that the bacterial collection was suitable to optimize and evaluate the models of virulence of S. anginosus.

**TABLE 1:** Phenotypic characterization of the clinical strains of S. anginosus used in the study

Strain	Source of isolate^*a*^	Lancefield antigen	Type of hemolysis	DNAse activity	Envelope
980/01	Blood	F	Gamma	+	Yes
4188/08	Pharyngitis	G	Beta	+	Yes
1658/06	Abscess	C	Alpha	−	Yes
4447/08	Abscess	G	Beta	+	Yes
4810/08	Pharyngitis	C	Alpha	+	Yes
2027/08	Abscess	A	Gamma	−	Yes
5652/09	Blood	C	Alpha	+	Yes
3114/96	Pharyngitis	C	Alpha	+	Yes
1834/01	Pharyngitis	C	Alpha	−	Yes
3027/03	Pharyngitis	C	Alpha	+	Yes
4020/05	Pharyngitis	F	Alpha	+	Yes
2087/07	Abscess	G	Alpha	+	Yes
2091/07	Pharyngitis	C	Alpha	+	Yes
2448/07	Skin wound	C	Alpha	+	Yes
2721/07	Skin wound	F	Gamma	−	Yes
3766/07	Abscess	C	Alpha	−	Yes
910/08	Blood	C	Alpha	+	Yes
4695/08	Pharyngitis	G	Beta	+	Yes
4737/08	Blood	No	Gamma	−	Yes
1505/09	Abscess	No	Gamma	−	Yes
1506/09	Abscess	No	Gamma	+	Yes
3792/10	Abscess	G	Beta	+	Yes
884/14	Pharyngitis	No	Beta	+	Yes

a Four strains were isolated from cases of bacteremia (blood), 8 from abscesses, 9 from cases of pharyngitis, and 2 from skin wounds.

### D. discoideum as a model organism for large-scale screening of S. anginosus virulence.

**FIG 1:**
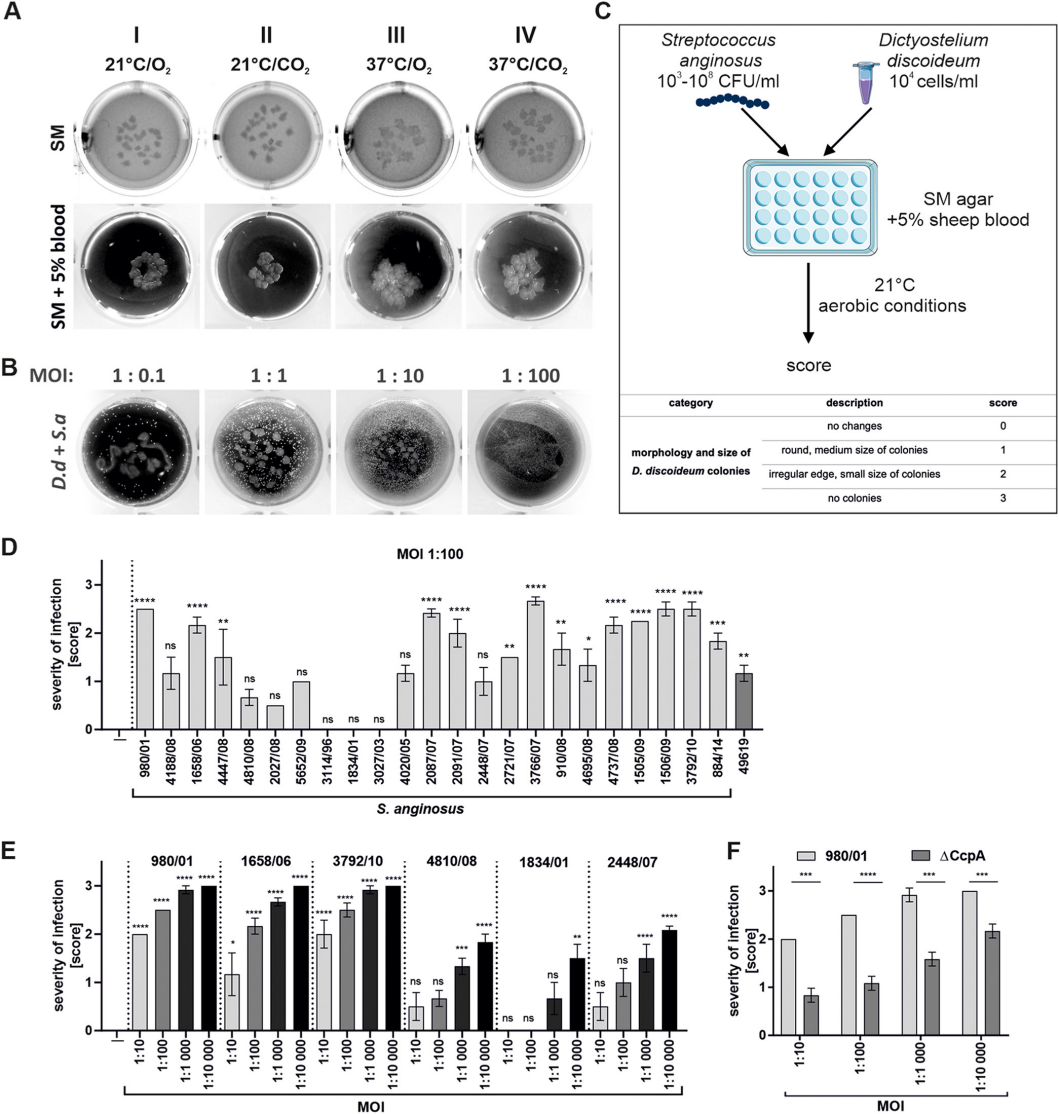
Infection of D. discoideum by S. anginosus. (A) Optimization of the growth conditions for D. discoideum. D. discoideum was seeded in SM and SM + 5% sheep blood medium at a density of 10^4^ cells/mL. Growth was carried out for 4 days under the following conditions: (I) 20% oxygen atmosphere, 21°C (21°C/O_2_); (II) 21°C, microaerophilic atmosphere enriched with CO_2_ (21°C/CO_2_); (III) 20% oxygen atmosphere, 37°C (37°C/O_2_); and (IV) 37°C, microaerophilic atmosphere enriched with CO_2_ (37°C/CO_2_). (B) Representative photographs of D. discoideum growth inhibition after coculture with S. anginosus 980/01 at MOIs of 1:0.1 to 1:100. (C) Protocol for adoption of D. discoideum as a model for studying the virulence of S. anginosus. (D) Quantification of D. discoideum deterioration after S. anginosus infection. S. anginosus was seeded in SM medium along with D. discoideum at an MOI of 1:100. Cultures were monitored for 5 to 10 days. D. discoideum plated on SM medium in the absence of bacteria was used as a control. After 5 days of coculture at room temperature under aerobic conditions, D. discoideum growth was assessed and scored for the number and morphology of colonies. (E, F) Dose-dependent pathogenic effects of S. anginosus strains (E) and comparison of severities of infection caused by the WT (980/01) and the ΔCcpA mutant (980/01 ΔCcpA) (F). Data represent mean values from three independent experiments ± standard errors of the means (SEM). *, *P* < 0.05; **, *P* < 0.01; ***, *P* < 0.001; ****, *P* < 0.0001; ns, not significant.

To examine the ability of the collected S. anginosus strains to affect test organisms, we first focused on selecting a simple, low-cost, low-labor, and fast model. Therefore, we selected D. discoideum as a cellular model to study pathogen-phagocyte interactions, as such a simple screening model has never been described for S. anginosus. Therefore, we needed to optimize the whole procedure, from coculture of bacteria with amoebae to proper qualification and quantification of the interaction with the host. First, we optimized coculture of D. discoideum with S. anginosus. We found that the recommended conditions for D. discoideum growth (21°C, standard medium [SM] agar, and aerobic atmosphere) were inadequate for growing S. anginosus, because 52% of the tested S. anginosus isolates did not grow in the blood-free medium. Therefore, we modified the coculture conditions to enable the growth of S. anginosus without affecting the growth of D. discoideum. For this purpose, D. discoideum was seeded in SM medium with or without supplementation with 5% sheep blood, and plates were grown under the following four conditions: (I) 21°C, 20% oxygen atmosphere; (II) 21°C, microaerophilic atmosphere of 5% CO_2_; (III) 37°C, 20% oxygen atmosphere; and (IV) 37°C, microaerophilic atmosphere of 5% CO_2_ (Fig. 1A). The optimization process showed that culture at 37°C changed the morphology of D. discoideum colonies significantly, as they became jagged, fuzzy, and more transparent (Fig. 1A); thus, this temperature was eliminated for future experiments. The growth of D. discoideum at 21°C in blood-supplemented medium was more compact than that in SM medium. Under microaerophilic conditions, the growth of D. discoideum was slightly slower than the growth under aerobic conditions (Fig. 1A). Therefore, since the growth of S. anginosus was limited in the absence of a blood additive, we chose SM medium supplemented with 5% sheep blood, an oxygen atmosphere, and 21°C as our conditions for growth. Subsequently, we optimized D. discoideum infection with S. anginosus strain 980/01, which was isolated from cases of bacteriemia and classified as highly invasive. Infection was carried out at multiplicities of infection (MOIs) of 1:0.1 to 1:100 (Fig. 1B). The results showed that D. discoideum growth was inhibited efficiently by bacterial infection at an MOI of 1:10, with the most distinct effect observed at an MOI of 1:100 (Fig. 1B). The growth of D. discoideum was scored according to the number and morphology of the colonies compared with those from noninfected cultures of D. discoideum. A score of 0 describes typical and unaffected growth of amoebae, while a maximum score of 3 represents complete inhibition of growth by bacteria. As a positive control, we used Streptococcus pneumoniae ATCC 49619. Thus, we successfully used D. discoideum as a simple model to study the virulence of S. anginosus. The details of the newly developed protocol are presented in Fig. 1C. Next, we used the above-described protocol to examine the entire collection of S. anginosus strains in terms of virulence. D. discoideum was infected with bacteria at MOIs of 1:10 to 1:10 000 (Fig. 1D, Fig. S1), after which significant differences in strain-dependent pathogenicity were observed among the tested strains. Furthermore, we found that the pathogenic effect depended on the bacterial dose (Fig. 1E, Fig. S1). Finally, to confirm the suitability of the D. discoideum model for S. anginosus virulence studies, we examined the course of infection using an attenuated strain (generated in the S. anginosus 980/01 background) devoid of the catabolite control protein A (CcpA) (ΔCcpA mutant), which is a global regulator that controls the expression of many virulence factors in streptococci, including toxins (42). This mutation in other streptococci was used previously as a tool to demonstrate differences in virulence in various animal models (43–45). We found a significant reduction in the severity of D. discoideum infection using the mutated S. anginosus strain (Fig. 1F). The cumulative results indicate that D. discoideum is a useful model for fast and reliable large-scale screening of S. anginosus pathogenicity, as it allows discrimination of strains into high- and low-virulence tiers. However, it might not be sensitive enough to study minute differences in virulence.

### G. mellonella as a model to study S. anginosus.

**FIG 2:**
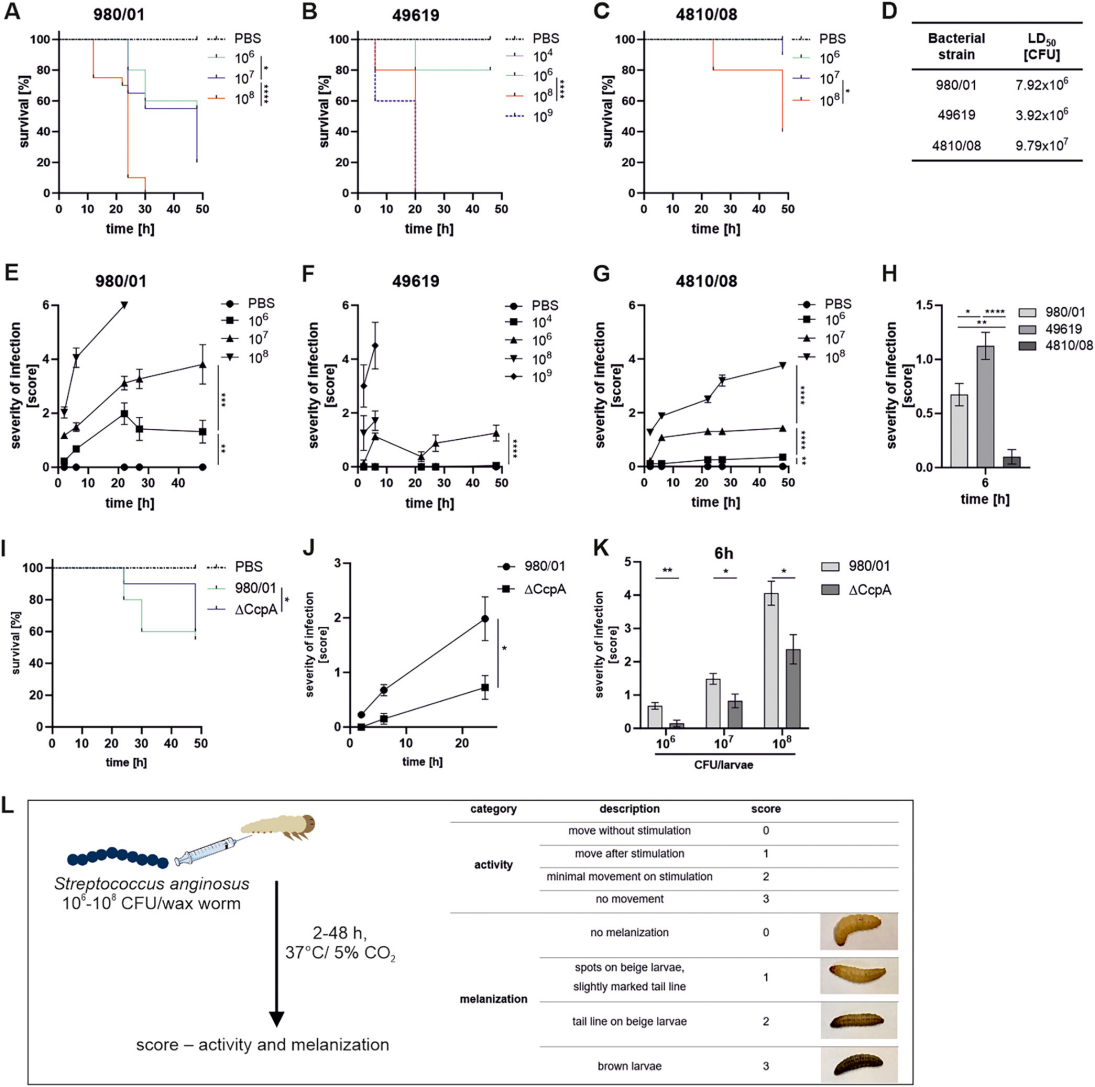
Virulence of S. anginosus and S. pneumoniae in G. mellonella larvae. Kaplan-Meier survival curves (A to C) and health index scores (E to G) of G. mellonella larvae after infection with different doses of S. anginosus and S. pneumoniae. The control group was injected with PBS. Each tested group contained 10 larvae (*n* = 10). (D) The LD_50s_ of S. anginosus and S. pneumoniae were calculated from a nonlinear regression analysis of larval death on day 2 p.i., with dose titration. Data are the mean values from two independent experiments. (H) Comparison of the degrees of severity of S. anginosus and S. pneumoniae infection at 6 h p.i. (10^6^ CFU/larva). (I) Kaplan-Meier survival curves of G. mellonella larvae infected with S. anginosus 980/01 and/or 980/01ΔCcpA. (J) The severity of infection was scored for 980/01 and/or 980/01 ΔCcpA (10^6^ CFU/larva). (K) The scores for larvae infected with different doses of 980/01 or 980/01 ΔCcpA were determined at 6 h p.i. (L) Protocol for adoption of G. mellonella larvae as a simple model for studying S. anginosus virulence. In all panels that include error bars, the data represent the mean values ± SEM. *, *P* < 0.05; **, *P* < 0.01; ***, *P* < 0.001; ****, *P* < 0.0001.

The experimental model based on G. mellonella has been used widely in recent years to study the mechanism underlying inactivation of innate immunity by pathogens like group A streptococcus (GAS), Streptococcus suis, and Streptococcus pneumoniae (46–50). However, G. mellonella has never been used to examine S. anginosus. Therefore, we decided to test whether the wax moth larva model could be used to study the strain-dependent pathogenicity of S. anginosus in more detail than the D. discoideum model. For this purpose, and in accordance with the results obtained from the D. discoideum model, we selected several strains of S. anginosus with various degrees of virulence (Fig. 1D). G. mellonella larvae were infected with one of the most (S. anginosus 980/01) and one of the least (S. anginosus 4810/08) virulent strains. S. pneumoniae ATCC 49619 was used as a positive control (48, 50). To determine the pathogenicity of S. anginosus in G. mellonella, a dose titration ranging from 1 × 10^6^ to 1 × 10^8^ CFU/larva was performed. Groups of 10 wax worms were infected through the last left proleg, with each larva receiving a single dose of S. pneumoniae, S. anginosus, or phosphate-buffered saline (PBS) (control). The highest pathogenic effect was observed in larvae infected with S. anginosus 980/01, which resulted in 100% mortality by 30 h postinfection (p.i.) at a dose of 1 × 10^8^ CFU/larva (Fig. 2A). In particular, the mortality kinetics resembled the effects obtained after infection with S. pneumoniae ATCC 49619 (Fig. 2B). Infection with S. anginosus 4810/08 showed lower mortality, as 80% of the larvae survived up to 24 h p.i. (Fig. 2C). Quantification of the lethal dose (50% lethal dose [LD_50_]) confirmed the significant differences in pathogenicity of the tested strains (Fig. 2D). Furthermore, wax worms were scored in terms of their melanization and activity (Fig. 2E to G). The data obtained revealed more severe symptoms in G. mellonella larvae infected with S. anginosus 980/01 (Fig. 2E) than in those infected with strain S. anginosus 4810/08 (Fig. 2G) at a dose of 1 × 10^7^ CFU/larva. When the larvae were infected with a dose of 10^6^ CFU, the severity of infection, estimated after 6 h, was significantly higher for strain 980/01 and S. pneumoniae than for strain 4810/08 (Fig. 2H). Furthermore, we found that the increase in the severity score and the decrease in survival were associated with the dose of bacteria. Next, we analyzed the pathogenic potential of the other S. anginosus strains in the collection. We found that strains isolated from cases of severe infection decreased larval survival (Fig. S2A) to a greater extent than isolates obtained from cases of mild infection (Fig. S2B). The ΔCcpA strain was used as a control infection. The data obtained showed a significant reduction in the mortality of larvae infected with the mutant strain compared to the mortality of larvae infected with the wild-type strain 980/01 (Fig. 2I). In particular, the severity of infection caused by 980/01 was significantly higher than that caused by the mutant (Fig. 2J) at 6 h p.i. and at all infectious doses tested (Fig. 2K). Thus, G. mellonella larvae are susceptible to infection by S. anginosus. The details of the newly developed protocol are presented in Fig. 2L.

### S. anginosus survives, proliferates, and disseminates in the larvae of Galleria mellonella.

**FIG 3:**
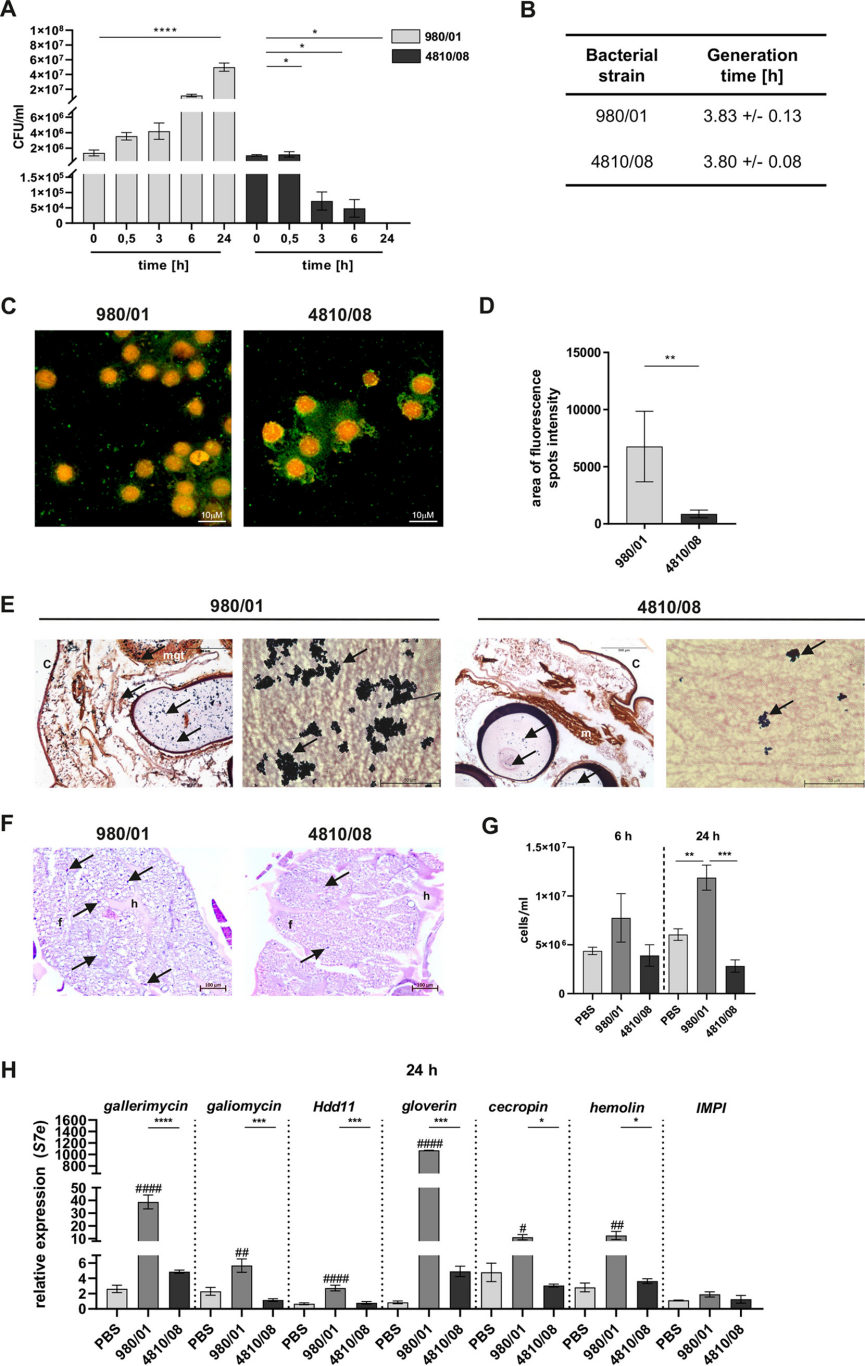
Survival of S. anginosus and activation of the innate immune response in G. mellonella larvae. (A) Larvae were infected with S. anginosus strains 980/01 and 4810/08 at a dose of 10^6^ CFU/larva. At each time point, individual larvae were homogenized in 1 mL of PBS and the viability of the bacteria was estimated by counting CFU after plating on blood agar. Each test group contained 10 larvae (*n* = 10). Error bars show SEM. *, *P* < 0.05; **, *P* < 0.01; ***, *P* < 0.001; ****, *P* < 0.0001. (B) The generation time, *G*, was calculated using the equation *G* = 0.301/*a*, where *a* is the slope of the linear part of the growth curve plotted on a semilogarithmic scale. The experiments were carried out in triplicate. Data represent the mean values ± standard deviations (SD). (C, D) LIVE/DEAD staining of S. anginosus and G. mellonella hemocytes after infection. The larvae were infected with 10^6^ CFU/larva. After 24 h, the larvae were sacrificed and the hemolymph was collected into a tube, seeded on polylysine slides, and stained with fluorescent LIVE/DEAD stain. (C) Viable cells of S. anginosus are stained green (SYTO 9), while red signals (propidium iodide [PI]) represent dead bacteria. Nuclear DNA from the host cells was stained with both SYTO 9 and PI. Scale bar = 10 μm. (D) Quantification of bacteria in the hemolymph of G. mellonella, calculated as the area of intensity of fluorescent spots from five different fields of view of the Z-stack. Error bars show SD. **, *P* < 0.01. (E) Gram-stained sections of G. mellonella larvae after S. anginosus infection (at 48 h p.i. with 10^6^ CFU/larva). Arrows indicate bacteria around tubular organelles. c, cuticle; m, muscle; mgt, midgut tissues. (F) Histological analysis of larvae (at 48 h p.i. with 10^6^ CFU/larva) was performed using hematoxylin-eosin staining (H&E). Arrows indicate areas of melanization. f, fat body; h, hemolymph. (G) Hemocytes of larvae (infected with bacteria at 10^6^ CFU/larva) were extracted, and they were counted using a Fuchs-Rosenthal chamber. Error bars show SEM. **, *P* < 0.01; ***, *P* < 0.001. (H) qRT-PCR analysis of G. mellonella gene expression 24 h p.i. The larvae were frozen in liquid nitrogen and then lysed with TRIzol. RNA was isolated, and qRT-PCR was performed. Relative expression levels of the gallerimycin, galiomycin, *Hdd11*, gloverin, cecropin, hemolin, and IMPI genes are shown. The gene encoding ribosomal protein S7e (a housekeeping gene) was used for normalization. Data represent mean values from three independent experiments ± SEM. *P* values indicated by pound signs (#) are for comparisons to the control. *#*, *P* < 0.05; ##, *P* <0.01; ###, *P* < 0.001; ###*#*, *P* < 0.0001; *, *P* < 0.05; **, *P* < 0.01; ***, *P* < 0.001; ****, *P* < 0.0001.

To identify factors that influence the deterioration of health status and mortality of G. mellonella larvae observed during S. anginosus infection, we examined the survival of bacteria in G. mellonella larvae. For that purpose, we applied a sublethal dose of S. anginosus (1 × 10^6^ CFU/larva) and monitored the number of bacteria at 0.5, 3, 6, and 24 h p.i. At each time point, an individual larva was homogenized in PBS, and the liquid obtained was plated. We found that the number of highly virulent S. anginosus 980/01 bacteria recovered from the lysates increased over time (Fig. 3A), while the number of low-virulence S. anginosus 4810/08 bacteria decreased significantly (Fig. 3A). In particular, the estimated *in vitro* generation times in culture medium for both tested strains were comparable (Fig. 3B). To verify the above-described data, the hemolymph of infected larvae (1 × 10^6^ CFU/larva) was isolated at 24 h p.i., cytospun, and stained with LIVE/DEAD stain to visualize bacteria. There was a higher number of live highly pathogenic S. anginosus 980/01 bacteria in hemolymph (Fig. 3C, green spots) than there was of low-virulence S. anginosus 4810/08 bacteria (Fig. 3C and D). Furthermore, Gram staining identified more aggressive dissemination of S. anginosus 980/01 to adjacent tissue, in contrast to the dissemination of strain 4810/08 (Fig. 3E). Taken together, these data indicate that highly virulent strains of S. anginosus are capable of growing and disseminating in a wax moth larva, likely because they are resistant to the host immune response.

Thus, we decided to examine the immune response of larvae to S. anginosus infection. Infection with S. anginosus 980/01 induced substantial increases in melanization and hemocyte infiltration, which were more intense than those observed for S. anginosus 4810/08 infection (Fig. 3F). Quantification of hemocytes in hemolymph collected at 24 h p.i. revealed their significant increase in S. anginosus 980/01 infected larvae compared to their number in noninfected and larvae infected with 4810/08 (Fig. 3G). To examine the activation of the defense system of G. mellonella after S. anginosus infection, we selected genes that play the most crucial role in immune defense and microbial clearance (51). Among them are genes encoding antimicrobial peptides (AMPs) (gallerimycin, galiomycin, gloverin, and cecropin) (52), hemolin, which acts as an opsonin (53), and a metalloproteinase inhibitor (IMPI) that protects the host from bacterial proteases (54), as well as *Hdd1*, which promotes the formation of nodules (55). We examined the expression of these molecules after infection with S. anginosus. For this purpose, larvae were infected with 10^6^ CFU of 980/01 and/or 4810/08 strains, and RNA was collected at 6 and 24 h p.i. The expression of the gallerimycin, galiomycin, *Hdd11*, gloverin, cecropin, hemolin and IMPI genes was evaluated using quantitative reverse transcription-PCR (qRT-PCR) (Fig. 3H, Fig. S3). Of the studied transcripts, we observed a significant change only in *Hdd11* at 6 h p.i. (Fig. S3), the level of which increased by up to 3.5-fold after infection with both S. anginosus strains. In contrast to 4810/08, strain 980/01 induced a significant increase in all transcripts at 24 h p.i., except for IMPI gene transcripts (Fig. 3H). Thus, S. anginosus significantly promotes the mobilization of innate immune mechanisms but remains resistant to their bactericidal activity, which allows its efficient proliferation and dissemination in G. mellonella larvae. The data obtained indicate corruption of the host defense system by this pathogen.

### Comparison of results from S. anginosus virulence models with those from a human phagocyte model.

**FIG 4:**
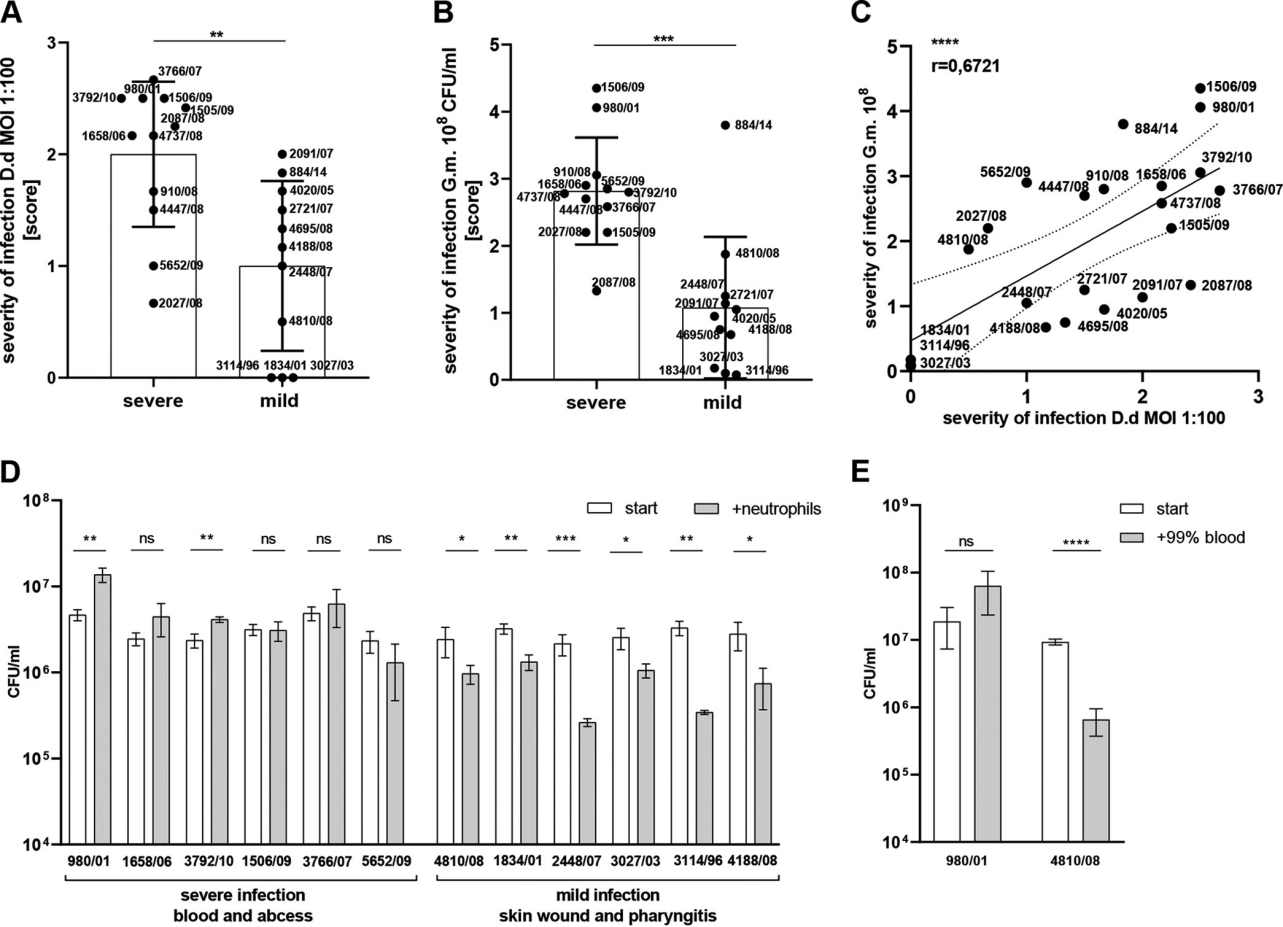
Comparison of the D. discoideum, G. mellonella, and human neutrophil models according to clinical severity of S. anginosus infection. (A, B) Comparison of the scores for severity of infection of D. discoideum (MOI of 1:100) (A) and G. mellonella (10^8^ CFU/larva) (B) with S. anginosus strains isolated from severe and mild infections. Error bars show SEM. (C) Pearson’s correlation analysis of the severity scores from the experiments whose results are shown in panels A and B. (D) Mean numbers of S. anginosus bacteria after a 90-min incubation with human neutrophils at an MOI of 1:5. After this time point, cells were lysed in 10% Triton X-100 and S. anginosus bacteria were counted after plating on blood agar. The experiments were carried out in duplicate. Data are presented as mean values ± SEM. (E) Killing of S. anginosus strains during incubation with whole blood. Suspensions of 10^7^ CFU/mL of S. anginosus strains were added to 99% human blood. After 6 h, bacterial survival was investigated. Error bars show SD. *, *P* < 0.05; **, *P* < 0.01; ***, *P* < 0.001; ****, *P* < 0.0001; ns, not significant.

Next, we compared the degrees of virulence of S. anginosus isolates in both nonmammalian models and examined the clinical significance of infection. We found a significantly higher severity of infection for isolates from blood and deep tissue abscesses than for isolates from skin wounds and upper respiratory tract infections in both D. discoideum (Fig. 4A) and G. mellonella (Fig. 4B). Furthermore, we confirmed that the pathogenicity observed in G. mellonella was consistent with the results obtained for D. discoideum, as reflected by a positive Pearson correlation coefficient (0.6721) (Fig. 4C); this suggests compatibility of the two infection models tested. To provide stronger evidence that the results obtained using D. discoideum and G. mellonella reflect those in humans, we used a model based on primary human neutrophils. We selected these leukocytes as the main components of innate immunity because they show phagocytic activity similar to those of D. discoideum (56, 57) and G. mellonella hemocytes (58). We then examined the bactericidal potential of neutrophils against selected strains (*n* = 12) from the collection. The data revealed that neutrophils significantly eradicated strains isolated from mild infections; however, strains isolated from bacteremia or abscesses were resistant (Fig. 4D). These results are consistent with the survival of bacteria in human blood (Fig. 4E). Collectively, the results indicate that the proposed models can be used to demonstrate the virulence potential of S. anginosus. Furthermore, our results suggest the pivotal role of phagocytes in defense against S. anginosus, which is reflected by the inactivation of bactericidal activity mediated by human neutrophils.

## DISCUSSION

The pathogenic potential of S. anginosus was only noticed recently by clinicians as a consequence of improved microbiological diagnostic methods (59). Data regarding the virulence of these streptococci are limited, and we have almost no data that explain the molecular mechanisms used by these microorganisms during the infection process. To investigate bacterial pathogenesis, we need to rely on well-established models of infection. The models must allow discrimination between high- and low-virulence strains and, at the same time, trace interactions with the defense mechanisms of the host. Because the interaction between the pathogen and the intact host immune system cannot be traced in *in vitro* models, researchers must rely on animal models of infection. According to the 3R recommendations for animal experiments (reduction, replacement, and refinement), we wanted to establish and optimize a nonmammalian model or models that will allow us to efficiently and rapidly screen the virulence properties of S. anginosus, with a special focus on the innate immune system and its reaction to infection. We wanted to show the benefits, as well as the limitations, of experiments using alternative models to assess the virulence of Streptococcus anginosus. Therefore, we chose two nonmammalian organisms: D. discoideum and G. mellonella.

The key to understanding the mechanism of virulence is identification of the components of the immune response that are affected by pathogens. Streptococcus anginosus is often isolated from abscesses, suggesting that leukocyte accumulation in response to the infection process is insufficient to eliminate the invaders. The role of leukocytes in the pathogenicity of bacteria belonging to the Streptococcus milleri group (the former name for the S. anginosus group) was noted, and its reduced eradication by phagocytes was observed (60). However, the mechanism by which it avoids phagocytes remains unexplained at the molecular level. Therefore, we focused our initial study on the phagocyte model of D. discoideum. This soil-dwelling amoeba has been used as a host for many important pathogens, including Vibrio cholerae (13), P. aeruginosa (61), and K. pneumoniae (62); however, the application of D. discoideum to the study of streptococci has barely been described. The only report comes from Bonifait et al., who documented that D. discoideum could be used to examine the virulence of S. suis (20). In our study, we carefully optimized the method of infecting D. discoideum with S. anginosus, showing that it could be applied to large screening analyses. Moreover, our data indicate that S. anginosus develops efficient strategies against phagocytes. Among them could be limited pathogen recognition and reduced engulfment by phagocytes. We should also consider the release of factors that can inactivate bactericidal activity or lead to cytolysis of host cells. The pathogenic effect mediated by extracellular and/or released bacterial compounds was observed in our pilot studies (Fig. S4). Therefore, the role of leukocidins, antiopsonins, and/or nucleases is a potential direction for further research that could explain why strains from more severe infections evade neutrophil killing better. The hypothesis of inactivation of phagocytes as a virulence mechanism of S. anginosus is strongly supported by the observations made using G. mellonella. Interestingly, despite significant increases in the production of chemotactic agents (e.g., hemolin), and in the influx, accumulation, and aggregation of hemocytes, we observed survival and multiplication of S. anginosus *in vivo*. It should be noted that there are many similarities between mammalian neutrophils and insect hemocytes (58). Insect hemocytes recognize pathogens and phagocytose them in a manner similar to that of neutrophils. Both human neutrophils and hemocytes have lectin-mediated phagocytosis and produce reactive oxygen species (ROS), extracellular traps, and antimicrobial peptides (AMPs). Additionally, both insect and human phagocytes possess the same receptors (e.g., Toll receptors), kinases (e.g., Jun N-terminal protein kinase [JNK]), and cascade pathways (e.g., JAK/STAT pathways). On the other hand, the molecular mechanisms used by D. discoideum to bind, ingest, and kill bacteria are analogous to those found in specialized phagocytic cells from multicellular organisms (56). Collectively, the inactivation of D. discoideum and of hemocytes of G. mellonella corroborates the resistance of S. anginosus to the bactericidal activity of human neutrophils, suggesting that these cells are the main target impaired by S. anginosus during infection. The above-described observation is of high importance and should be studied in more detail with the emphasis on the molecular mechanism underlying bacterial resistance to neutrophils.

The similarity of the innate response of G. mellonella larvae to that of mammalian systems allowed us to examine the mechanism through which humoral factors like AMPs work. Galleria mellonella produces a multitude of AMPs that are crucial for eliminating bacterial infection (30). Haine et al. proposed that these molecules play a more important role in counteracting persistent infection than in fighting acute infection (63). This could explain the delay in the expression of AMPs, except IMPI, that we documented 24 h after infection. The data obtained corroborate the observation made by Sheehan et al., who described an increase in gloverin and cecropin 24 h after infection with S. aureus (64). Furthermore, the increased expression of hemolin, a member of the immunoglobulin subfamily of recognition molecules (40), could help in the future to discriminate between lipoteichoic acids (LTAs) of S. anginosus, as the level of this protein depends on the LTA (65). These aspects could be investigated further, since putative regions encoding LTA have been identified in the S. anginosus genome, although their role in the pathogenesis of bacteria remains unknown (3).

G. mellonella seems to be a suitable model not only for studying the mechanisms underlying immune responses, but also for identifying the role of potential virulence factors. We documented this possibility by showing significant attenuation of strains devoid of CcpA. CcpA allows streptococci to absorb sugar from the environment, to colonize the oropharynx and nasopharynx, and to form biofilms (66–68). Furthermore, CcpA directly regulates the expression of toxins like streptolysin and intermedilysin by Streptococcus pyogenes and S. intermedius, respectively (42, 69, 70). Similarly, the application of the G. mellonella model to the S. anginosus study opens up the possibility of comparing S. anginosus with other streptococci, as in its previous use to examine S. pneumoniae (48, 50), Streptococcus agalactiae (71, 72), S. pyogenes (46, 47, 73, 74), and Streptococcus mutans (75, 76). A clear example is that our histological analysis revealed robust activation of immune defenses, which in some cases correlated with tissue damage, a common phenomenon associated with group A streptococcus infection (47). In addition to cytolysin genes, numerous putative genes that encode proteases have been identified in the genome of S. anginosus (3); thus, the role of these enzymes in disseminating bacteria from the hemolymph should be studied in the future.

The main limitation of using D. discoideum is temperature, as according to the optimized method, the experiments should be carried out at 21°C. Streptococci, including GAS (77), S. agalactiae (78), and S. pneumoniae (79), change their transcriptome profiles significantly when growing at temperatures lower than 37°C. GAS manifest significant decreases in hemolytic and nuclease activities when grown at 30°C. In contrast, some genes, mainly those involved in metabolism, replication, and recombination and in DNA repair, transport, and binding, are upregulated at lower temperatures (77). Therefore, S. anginosus growth and gene expression should be evaluated at the temperature used in the D. discoideum model when the molecular mechanism of the observed interaction is studied. This aspect can be omitted from the G. mellonella model, as it can be handled at a physiological mammalian temperature of 37°C (80, 81), giving it an apparent advantage over other invertebrate model hosts, such as Caenorhabditis elegans and Drosophila melanogaster (82). It should be noted that, as the G. mellonella genome has been described (41), we also have the possibility of genetic manipulation of the host. Furthermore, although we have not examined it for this, we propose that G. mellonella would be a suitable model for studying the efficacy of therapeutic agents *in vivo*, as described for other streptococci (50).

In conclusion, we show that D. discoideum and G. mellonella are suitable models for studying the pathogenicity of S. anginosus. The main advantage of the sequential application of both models presented herein is the ease of establishment and maintenance, the low cost, and the feasibility of high-throughput studies (72). The results obtained for both models are comparable, they corroborate clinical data, and the model based on human leukocytes allows the reliable selection of strains with extremely high or extremely low virulence.

## MATERIALS AND METHODS

### Bacterial strains.

S. anginosus strains were obtained from National Medicines Institute, Warsaw, Poland (83), while the Streptococcus pneumoniae control strain (ATCC 49619) was purchased from ATCC. Streptococci were grown in tryptic soy broth (TSB; Sigma-Aldrich) liquid medium at 37°C under microaerophilic conditions with 5% CO_2_. Bacterial cells were collected by centrifugation after overnight culture (5,000 × *g* for 5 min at 20°C), washed twice with PBS (Dulbecco’s phosphate-buffered saline without Ca^2+^ and Mg^2+^), and resuspended in PBS to an optical density of 1.0 measured at 600 nm (OD_600_), which corresponds to 1 × 10^8^ CFU per mL (CFU/mL).

### Construction of ΔCcpA strain.

The mutant construction (980/01 ΔCcpA) was performed in a manner analogous to the previously described construction of a Δ*codY* mutant (84). Primers were designed for the sequences upstream and downstream from both flanks of the *ccp*A gene (*ccpA* Left flank Forward, CTGTCCGTGTCATATCGCTGGCATAACC, and Left flank Reverse GTTATAGTTATTATAACATGTATTCCCGGGCATGCTTCTTCCTTTCTATATTGAAAATATCGTTTTCACATTC; *ccpA* Right flank Forward, TTAAATAACAGATTAAAAAAATTATAACCCGGGTAAGTAGAGTTAGACAGAACTTGAAATTTTCAATTTTAAG, and Right flank Reverse GCACCACAATCCCTTCTGTTTCTTCATAACTG) and for the spectinomycin resistance gene from plasmid pSL60 (85) (*spc* forward CCCGGGAATACATGTTATAATAACTATAACTAAT, and *spc* reverse, CCCGGGTTATAATTTTTTTAATCTGTTATTT).

### Phenotypic characterization of S. anginosus strains.

The Lancefield antigen detection was performed using the streptococcal grouping kit (Oxoid) according to the manufacturer’s instructions. Qualitative determination of hemolysis was performed after 24 h of bacterial growth in Columbia agar medium (Sigma-Aldrich) with 5% sheep blood. The appearance of a clear zone surrounding the colony was classified as beta-hemolysis, while a greenish zone was classified as alpha-hemolysis and no zone was designated as gamma-hemolysis. DNase activity was examined by plating bacteria on BD DNase test agar plates. Plates were incubated at 37°C under microaerophilic conditions with 5% CO_2_ for 24 h. After incubation, 1 M HCl was poured onto the plate and incubated for at least 2 min. On the basis of the presence of a clear zone around the bacteria, the ability to produce DNases was determined. The presence of a cell capsule was estimated by negative dyeing with India ink and visualization under a light microscope.

### Growth properties of S. anginosus strains.

S. anginosus strains were transferred from plates with Columbia agar with 5% sheep blood to a TSB medium and grown at 37°C under microaerophilic conditions with 5% CO_2_. After overnight culture, bacteria were used to inoculate new cultures at an OD_600_ of 0.15 in duplicates and grown under the same conditions. The optical density of the culture was measured at a wavelength of 600 nm every 2 h for 10 h. Generation times, *G*, were calculated with the equation *G* = 0.301/*a*, where *a* is the slope of the linear part of the growth curve.

### Culture of D. discoideum.

D. discoideum ATCC 28368 was grown in glass flasks with liquid medium HL-5 supplemented with glucose, vitamins and microelements (Formedium, England - cat# HLE1) (14 g/L peptone, 7 g/L yeast extract, 13.5 g/L glucose, 0.5 g/L KH_2_PO_4_, 0.5 g/L Na_2_HPO_4_, 0.01 g/L FM vitamins, and microelements) at 21°C with shaking (180 rpm) until used for experiments. Cell density was monitored by cell counting using a Fuchs-Rosenthal chamber. Cell density in culture did not exceed 10^7^ cells/mL. For infection studies, cells were counted and diluted to a density of 10^4^ cells/mL and seeded in solid medium. The culture method was optimized as described in Results.

### Bacterial virulence assay with D. discoideum.

Overnight cultures of S. anginosus or control strains were diluted in PBS to the desired serial densities of 10^3^ to 10^8^ CFU/mL. Fifty microliters of each S. anginosus dilution or control strain was deposited in a well of a 24-well plate filled with 2 mL of 5% sheep blood SM agar (10 g/L peptone, 1 g/L yeast extract, 10 g/L glucose, 1.9 g/L KH_2_PO_4_, 1.3 g/L K_2_HPO_4_ 3H_2_O, 0.49 g/L anhydrous MgSO_4_, 16 g/L agar). When the surface of the well was dry, five μL of HL-5 medium containing 10^4^ cells/mL of D. discoideum was added to the bacterial lawn and the plate was incubated at 21°C.

To discriminate the effect of bacterial phagocytosis from the activity of secreted bacterial compounds, D. discoideum was incubated with 5 μM cytochalasin D for 30 min at room temperature before being added to the bacterial lawn (86). The growth of D. discoideum with S. anginosus was monitored for 5 to 10 days. After 5 days of coculture, the inhibition of D. discoideum growth was scored for the number and morphology of D. discoideum colonies and compared to the growth of D. discoideum seeded alone. The score representing typical, unaffected growth of D. discoideum was set at 0, and changes in morphology were addressed as presented in Fig. 1. If necessary, intermediate values were used.

### G. mellonella infection model.

The G. mellonella larvae were purchased from Biosystems Technology and stored in the dark at 17°C. Only healthy larvae with no signs of melanization were used in the experiments. Ten larvae per group were infected by injection into the last proleg using a Hamilton syringe with 10 μL of bacterial inoculum containing from 10^5^ to 10^8^ CFU of S. anginosus. Ten microliters of PBS was used as a control. The larvae were incubated at 37°C in 9-cm Petri dishes without food. Their condition in terms of activity and melanization was monitored up to 48 h after infection and scored as presented in Fig. 2. The larvae were considered dead when they did not move in response to tactile stimulation.

### Isolation of the hemolymph of G. mellonella.

To collect larval hemolymph, larvae were wiped with 70% ethanol and then an incision was made on the proleg with a scalpel. Five microliters of hemolymph was mixed with the same volume of ice-cold PBS buffer with 0.36% β-mercaptoethanol to prevent coagulation and melanization and immediately used for the quantification of the number of hemocytes by trypan blue staining.

### Bacterial viability in the hemolymph of G. mellonella.

The survival of bacteria in hemolymph was examined using the LIVE/DEAD BacLight kit (Molecular Probes). The hemolymph was collected as described above and cytospun on polylysine glass. The staining procedure was performed according to the manufacturer’s instructions. Bacteria were visualized by confocal laser scanning microscopy (CLSM) using a Zeiss LSM 880 confocal system equipped with 100× oil immersion objectives. The acquired Z-stack images from five different fields of view were analyzed using Zeiss ZEN microscopy software and Fiji software. The quantification of bacteria in the hemolymph of G. mellonella was calculated as an area of fluorescence spot intensity using ImageJ software.

### Bacterial survival/proliferation in G. mellonella larvae.

At different time points postinfection (p.i.) of G. mellonella larvae with S. anginosus, larvae were homogenized in 1 mL of PBS by mechanical force. The samples were serially diluted in PBS and plated on Columbia agar with 5% sheep blood, and colonies were counted after incubation at 37°C for 48 h. As a control, PBS-injected larvae were used in an analogous procedure.

### Histopathological analysis of G. mellonella specimens.

Larvae injected with 10 μL of PBS (control) or 10^6^ CFU of S. anginosus were sacrificed by freezing 48 h after injection. The larvae were then injected with 100 μL of 10% formalin to fix internal organs and then stored for 24 h at 4°C. The larvae were cut along segments and embedded into wax blocks. Tissue sections (10 μm) were stained with hematoxylin-eosin or Gram stain. Sections were examined using a light microscope.

### RNA isolation and qRT-PCR.

Six and 24 h after infection, larvae infected with 10^6^ CFU of S. anginosus were snap-frozen in liquid nitrogen and homogenized. Then, TRI Reagent (Sigma-Aldrich) was used to extract RNA according to the manufacturer’s instructions. Reverse transcription was performed using the high-capacity cDNA reverse transcription kit (Applied Biosystems). One microgram of RNA from each sample was used for cDNA synthesis with oligo(dT) primers according to the manufacturer’s instructions. Quantitative reverse transcription-PCR (qRT-PCR) was performed with the SYBR green method in a reaction mixture volume of 15 μL, containing 0.5 μL of cDNA sample, 10 μM forward and reverse primers, and 1× GoTaq qPCR master mix (Promega). The primers and conditions for denaturation, annealing, and extension for each pair of primers are listed in Table S1 (53, 65, 87, 88).

The qRT-PCR was initiated by denaturation for 3 min, and the amplification program was carried out for 44 cycles with a final elongation step at 72°C for 10 min.

The gene encoding ribosomal protein S7e, a housekeeping gene, was used for normalization. The means of the threshold cycle (*C_T_*) values were calculated and analyzed using the ΔΔ*C_T_* quantification method (89). To verify the specificity of quantitative PCR (qPCR) products, melt curve analyses were performed.

### Isolation of human neutrophils.

Blood was purchased from the Regional Blood Center, Krakow, Poland. It was collected from healthy donors who provided written informed consent for the collection of samples and subsequent cell isolation and analysis. For human subject confidentiality assurances, blood material was de-identified; thus, this study adheres to appropriate exclusions from human subject approval. Neutrophils were isolated from fractions of peripheral blood enriched with granulocytes, which were harvested using a density gradient. Neutrophils and erythrocytes were collected as the high-density fraction. To separate neutrophils from erythrocytes, this fraction was incubated for 30 min with 1% polyvinyl alcohol (POCH; Poland). Neutrophils were collected from the upper layer. After centrifugation (280 × *g* for 10 min at 20°C), other erythrocytes were removed by lysis in water. Neutrophils were resuspended in serum-free Dulbecco modified Eagle medium (DMEM) without phenol red (Gibco/ThermoFisher Scientific, USA).

### PMN killing assay.

A suspension of S. anginosus in DMEM (without phenol red) with 1% autological human serum was added to 5 × 10^5^ polymorphonuclear leukocytes (PMN)/well seeded in DMEM in 96-well plates at an MOI of 1:5 (cells-to-bacteria ratio), and the plates were centrifuged (300 × *g* for 8 min at 20°C). PMN and S. anginosus were cocultured for 90 min in a humidified atmosphere containing 5% CO_2_. After 90 min, cells were lysed by adding 20 μL of 10% Triton X-100. The samples were serially diluted in PBS and seeded in Columbia agar with 5% sheep blood, and colonies were counted after incubation at 37°C for 48 h.

### Whole-blood killing.

A suspension of 10^7^ CFU/mL of S. anginosus was added to human blood. The samples were incubated for 6 h at 37°C. After 6 h, samples were serially diluted in PBS and plated on Columbia agar with 5% sheep blood, and colonies were counted after incubation at 37°C for 48 h.

### Statistical analysis.

Data were analyzed using GraphPad Prism version 9.1.1 (GraphPad Software). Parametric tests (unpaired Student’s *t* test or analysis of variance [ANOVA] and Pearson correlation coefficients) were used. A *P* value of <0.05 was used for statistical significance. The logarithmic rank test (Mantel-Cox) was used for survival analyses.
