# Supplementary material for: Studies of Streptococcus anginosus Virulence in Dictyostelium discoideum and Galleria mellonella Models
Source: Infect Immun. 2023 Apr 25;91(5):e00016-23. doi: 10.1128/iai.00016-23 (PMC10187118; doi:10.1128/iai.00016-23)
Supplement: Supplemental file 1 — Fig. S1 to S4 and Table S1. Download iai.00016-23-s0001.pdf, PDF file, 0.7 MB [file iai.00016-23-s0001.pdf]

Supplement fig. S1

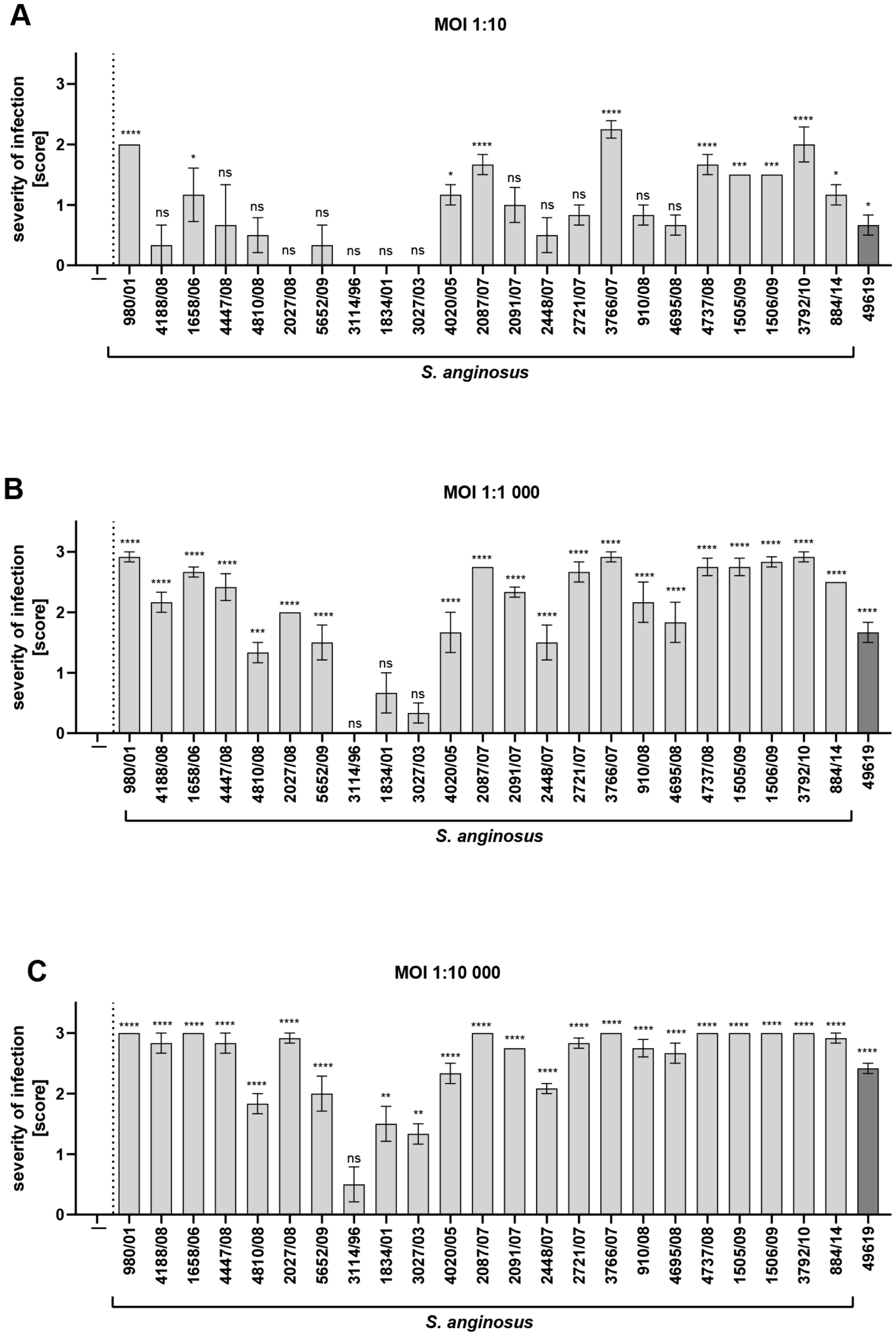

Supplement fig. S2

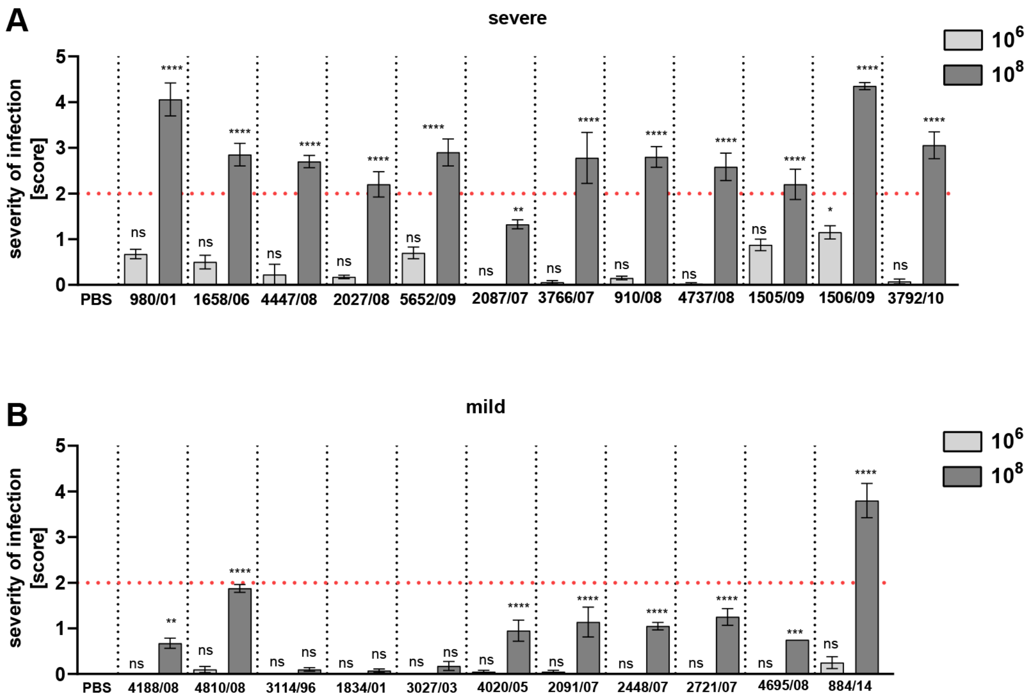

Suplement fig. S3

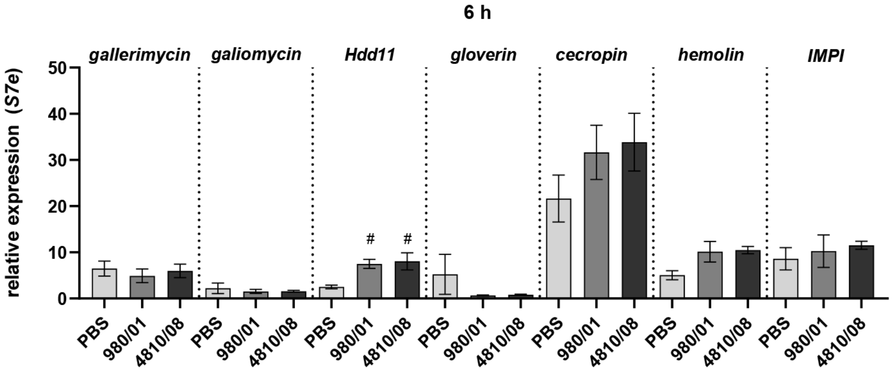

Supplement fig. S4

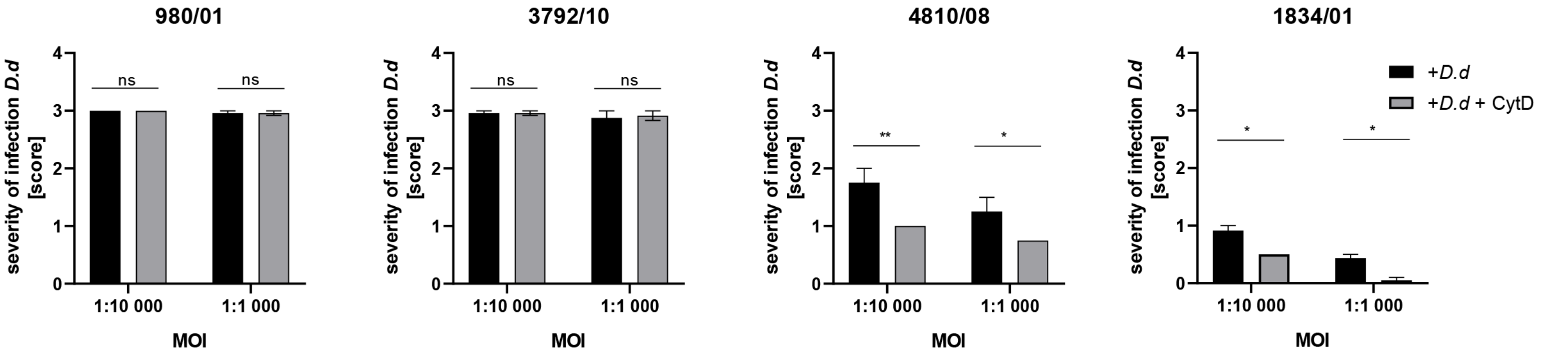

Table S1. Oligonucleotide sequences used in quantitative reverse transcription-PCR (qRT-PCR).

Oligonucleotide	Sequence	ref	Temperature program[1]
**S7e forward F**	5'-ATGTGCCAATGCCCAAGTTG-3'	(87)	1. 95˚C, 15 s<br>2. 60˚C, 15 s<br>3. 72˚C, 1 min
**S7e reverse R**	5'-GTGGCTAGGCTTGGGAAG AAT-3'		
**gallerimycin F**	5'-TATCATTGGCCTTCTTGGCTG-3'		
**gallerimycin R**	5'-GCACTCGTAAAATACACATCCGG-3'		
**galiomycin F**	5'-TCGTATCGTCACCGCAAAATG-3'		
**galiomycin R**	5'-GCCGCAATGACCACCTTTATA-3'		
**gloverin F**	5'-GTGTTGAGCCCGTATGGGAA -3'	(53)	
**gloverin R**	5'-CCGTGCATCTGCTTGCTAAC-3'		
**cecropin F**	5'-CTGTTCGTGTTCGCTTGTGT-3'		
**cecropin R**	5'-GTAGCTGCTTCGCCTACCAC-3'		
**hemolin F**	5'-CTCCCTCACGGAGGACAAAC-3'		
**hemolin R**	5'-GCCACGCACATGTATTCACC-3'		
**IMPI F**	5'-TAGTAAGCAGTAGCATAGTCC-3'	(88)	
**IMPI R**	5'-GCCATCTTCACAGTAGCA-3'		
**Hdd11 F**	5'-TCGGCTTGTGAGTTCGTTGT-3'	(65)	1. 95˚C, 15 s<br>2. 58˚C, 15 s<br>3. 72˚C, 1 min
**Hdd11 R**	5'-GGCACTAGAAGGAGCACCAC-3'		

[1]Denaturation (1); annealing (2); extension (3).

Supplemental Figure Legends

Fig. S1 Inhibition of the growth of *D. discoideum* induced by *S. anginosus.* *S. anginosus* was seeded in SM medium along with *D. discoideum* at an MOI of **(A)** 1:10, **(B)** 1:1 000, or **(C)** 1:10 000. Cultures were carried out for 10 days at room temperature under aerobic conditions. *D. discoideum* plated on SM medium without bacteria was used as the control. After 5 days of co-culture, the growth of *D. discoideum* was assessed both alone and in the presence of bacteria, and growth was scored according to the number and morphology of colonies. Data represent mean values of three independent experiments ± SEM (*, P < 0.05; **, P < 0.01; ***, P < 0.001; ****, P < 0.0001; ns, not significant).

Fig. S2 Use of the *G. mellonella* model to assess the virulence of different strains of *S. anginosus* isolated from severe (A) and mild (B) clinical infections. Health index scores of *G. mellonella* larvae (infected with 10^6 and 10^8 CFU/larva) after 6 h p.i. The control group was injected with PBS. Each tested group contained 10 larvae (n = 10). The values represent the mean ± SEM (*, P < 0.05; **, P < 0.01; ***, P < 0.001; ****, P < 0.0001; ns, not significant).

Fig. S3 Activation of innate immune response in *G. mellonella* infected with S. anginosus strains. qRT-PCR analysis of *G. mellonella* gene expression was measured at 6 h.p.i. The larvae were frozen in liquid nitrogen and then lysed in TRIzol. RNA was isolated and qRT-PCR was performed. Relative expression of *gallerimycin*, *galiomycin*, *Hdd11*, *gloverin*, *cecropin*, *hemolin*, and *IMPI* is shown. The ribosomal protein *S7e* (a housekeeping gene) was used for normalization. Data represent mean values from three independent experiments ± SEM (#P<0.05, vs Control).

Fig. S4.

Quantification of *D. discoideum* deterioration after *S. anginosus* infection in the presence of Cytochalasin D. *S. anginosus* was seeded in SM medium along with *D. discoideum* with or without Cytochalasin D (5 µM) at an MOI of 1:10 000 and 1:1 000. Cultures were monitored for 5 to 10 days. *D. discoideum* plated on SM medium in the absence of bacteria was used as a control. After 5 days of co-culture at room temperature under aerobic conditions, *D. discoideum* growth was assessed and scored for the number and morphology of colonies. Data represent mean values of two independent experiments ± SEM (*, $P < 0.05$; **, $P < 0.01$; ns, not significant.).

Table S1. Oligonucleotide sequences used in quantitative reverse transcription-PCR (qRT-PCR). [1]Denaturation (1); annealing (2); extension (3).
